# Implementing part-time leadership as instrument for sustainable HR management

**DOI:** 10.1186/s40991-020-00053-3

**Published:** 2020-12-21

**Authors:** Anja Karlshaus

**Affiliations:** CBS International Business School, Hardefuststraße 1, 50677 Köln, Germany

**Keywords:** Part-time leadership, Atypical working time arrangements, Working time models, Sustainable HRM, Triple bottom line, Sustainable development goals

## Abstract

This paper discusses the suitability of part-time leadership as instrument for a sustainable Human Resources Management (HRM) policy. The concept of part-time leadership is introduced and discussed based on a meta-analysis of existing studies and latest research that has been executed on‚ atypical‘working time arrangements in leadership positions. The article discusses the intersection of part-time leadership with the three subject areas (economic, social and ecological) of the Triple-Bottom Line and tries to show more specifically how part-time leadership models can serve the fulfillment of selected Sustainable Development Goals (SDGs), especially in Western countries. Finally, prerequisites and common barriers for a successful implementation of part-time leadership as Corporate Social Responsibility (CSR) instrument of organizations are unveiled, and suggestions for mitigating those challenges are given from a macro, micro and process perspective.

## Introduction

Time or more precise ‘lack of time’ are identified as one of the biggest problems for families in Germany (BMFSFJ (Bundesministerium für Familie, Senioren, Frauen und Jugend), 2017; Familienbericht, 2015), and one can assume transferability of this finding to other developed countries. Present studies highlight the desire for fewer working hours on all levels including management, along with the increasing importance of part-time leadership models (Karlshaus, 2016; Karlshaus & Kaehler, 2017a).

The data of the DIW (Deutsches Institut für Wirtschaftsforschung) 2015 shows that the favored working time per week for women in leading positions amounts to only 34 h, for men it amounts to 38 h (Karlshaus & Kaehler, 2017b). Therefore, one could expect that the majority that desires part-time leadership models is female. However, the *2nd Väter-Barometer* (fathers’ barometer) in 2016 and the *Väterreport* (fathers’ report) of the government in 2018 (BMFSFJ (Bundesministerium für Familie, Senioren, Frauen und Jugend), 2018) showed that also 51% of employed fathers would like to work less in order to have more family-time (Karlshaus & Kaehler, 2017c). On that account, ‘working time’ has been defined as one of the three central pillars of family policy in Germany (Kolat & Schirmacher, 2017).

However, this topic has not only reached society, media and family policy – it is also greatly popular from a company perspective. Several organizations have already implemented part-time leadership programs and even more offer individual models ‘on demand’. These examples demonstrate that flexible working models are not only desired – they might also serve company interests and seem to work well particularly for lower and middle management positions (Karlshaus, 2016; Karlshaus, 2018). Global trends such as demographic change and the resulting shortage of skilled labor in some sectors, the ageing workforce and the increasing retirement age with respected health related issues – as well as a value shift towards balance issues, especially among generation Y, force companies to rethink traditional full-time leadership models and to tackle more flexible working time models (Karlshaus, 2018; Karlshaus & Kaehler, 2017a).

At the same time the popularity and importance for corporate social responsibility (CSR) has increased in the business world. Many corporations in Germany already have well established CSR departments and are familiar with respect to the relevant national and international standards (Lindgreen & Swaen, 2010). The 17 sustainable development goals (SDGs) developed and adopted by all 193 member states of the United Nations in 2015 have not just led to to an increased focus on CSR in the business and scientific community – but also to the formulation of specific global goals and herewith a framework to action, which has been operationalized in 169 subgoals (United Nations, 2018).

Even though both ‘part-time leadership’ and ‘CSR’ have gained acceptance and importance to some extent and share comparable objectives, there are no research studies linking these two concepts. Understanding part-time leadership as contributor and driver of sustainability thereby offers a framework for theoretical discussion of how part-time leadership might serve as instrument for a sustainable HR management while supporting selected SDGs at the same time. Beyond that, implications for the extended and sustainable use of part-time leadership models in business might be derived. Being aware of the benefits of part-time leadership in the context of a sustainable HR management approach and to also fulfill individual SDG indicators might further challenge potential concerns about management positions with atypical working time arrangements and highlights the needs of organizations to reconsider this concept.

In response to the depicted developments and trends with respect to sustainability needs and the desire for more flexible working time arrangements on all management levels, this article tries to answer to what extent part-time leadership constitutes an instrument of a sustainable HR policy and how it might serve the ‘Sustainable Development Goals’. Therefore, in the following, the technical terms ‘CSR’, ‘Sustainable HRM’ and ‘part-time leadership’ are defined and brought together by using the ‘Triple Bottom Line’ framework as an example. Building on this analysis, a more specific application of the benefits of ‘part-time leadership’ with respect to the fulfillment of SDGs (especially in Western countries) is given. This requires a systematic implementation of part-time management, which will be presented in the following chapter. The article ends with a critical discussion of the benefits and limitations of part-time leadership as instrument for sustainable HRM.

## Understanding of CSR and sustainable HRM

In the following chapter, critical explanations of the terms and concepts ‘Corporate Social Responsibility’ (CSR) as well as ‘Sustainable Human Resource Management’ will be presented.

### Definition of corporate social responsibility

Nowadays the concept of Corporate Social Responsibility (CSR) is not just omnipresent – there is a multitude of further nomenclatures for everyday use, like e.g. ‘Sustainability’, ‘Corporate Sustainability‘, ‘Corporate Citizenship‘, ‘Corporate Accountability‘, or ‘Business Ethic‘. Even in scientific literature the classification and delimitation of the various terms is not absolutely selective (Keinert, 2008; Schneider, 2012; Seidel, 2011). Furthermore, even within the existing definitions of CSR there are significant inconsistencies and divergent foci points (Dahlsrud, 2006; Duong Dinh, 2011; Schneider, 2012), as has been aptly expressed by Votaw und Sethi (1973, p. 11; cf. Schneider, 2012, p. 18): „The term is a brilliant one, it means something, but not always the same thing to everybody. To some it conveys the idea of legal responsibility or liability; to others it means socially responsible behavior in an ethical sense; to still others, the meaning transmitted is that of ‚responsible for‘, is a casual mode; many simply equate it with a charitable contribution.”

Nevertheless, the definitions of the European Commission from 2001 and 2002 are relatively common and accepted in practice for European companies (European Commission, 2001, p. 7): „A concept whereby companies integrate social and environmental concerns in their business operations and in their interactions with stakeholders on a voluntary basis.” Likewise, the definitions from the years 2011 resp. 2014 (European Commission, 2011, p. 6): „the responsibility of enterprises for their impacts on society “as well as “… process to integrate social, environmental, ethical and human rights concerns into their business operations and core strategy in close interactions with their stakeholders”.

The fact, that there is no single, universally accepted definition of CSR, can be considered as less problematic as there are certain contents that are largely congruent like e.g. the description of the central CSR dimensions (Dahlsrud, 2006). Against this background, Loew, Ankele, Braun, and Clausen (2004) identified the following common attributes out of the range of definitions and discussions around CSR:

- CSR typically addresses the ecology, economy and society
- CSR includes compliance with all relevant legislations – but concentrates beyond that on ‘corporate engagement’
- CSR intends to contribute to ‘sustainable development’

Research with respect to Corporate Sustainability typically centers around the question, how business might work ‘different’ in future times. One important insight is, that ‘sustainable development’ cannot be realized without the support of companies (Bansal, 2005). A second important realization is the fact, that corporate sustainability is gaining increasing strategic relevance for companies due to globalization, humanitarian and ecological catastrophes, (financial) scandals, and demographic shifts (Ehnert, 2012). Considering such a business environment and resulting corporate challenges, a third realization is becoming obvious: Sustainability as well as CSR is strongly affected by HRM and affects HRM at the same time (Ehnert, 2009; Jabbour & Santos, 2008; Zaugg, 2009).

### Definition of sustainable human resource management

As described in the previous section, the increasing strategic relevance of sustainability for HRM is the result of adding social (Zaugg, 2009) and ecological (Jabbour & Santos, 2008) components to the core strategy of many multinational companies. Furthermore, the necessity of analyzing and managing regeneration- and reproduction-conditions of the workforce due to skill shortages intensifies the role of HRM in that its visibly adds value to the sustainable development of corporations (Ehnert, 2009; Zaugg, 2009), requiring a paradigm shift with respect to traditional HRM approaches (Ehnert, 2012).

Within the last 20 years many researchers have analyzed the relationship between CSR and HRM (e.g. Clarke, 2011; Ehnert, 2009; Ehnert, 2012; Pfeffer, 2010; Zaugg, 2009). Even though the approaches sometimes differ strongly with respect to the underlying CSR definition as well as with respect to scope and disciplinary background, there is some consensus that HR researchers and specialists are especially sensible towards the underlying CSR concept. There are similar considerations of CSR and HRM concerning long-term resource balancing, efficiency of (human) resources, and development and prevention of (human) resources (Ehnert, 2012; Müller-Christ, 2010). In this context some authors indeed distance themselves from the controversial designation of ‘human beings’ as resources (Ehnert, 2009) and suggest avoiding viewing employees as cost and production factor that is aimed to be minimized – but rather to perceive the workforce as one of the most valuable resources for a business which has to be cared for, developed and promoted (Greenwood, 2002).

However, remaining in the metaphor of ‘resources’, there is some consensus that ‘human resources’ might run short in certain job functions, which is reflected by current and increasingly intensive discussions about the lack of a skilled workforce. A pure focus on recruiting efforts might not be sufficient to deal with the expected long-term lack of talents (Ehnert, 2012). Following a sustainability perspective on HRM, further conditions like family and personal environment need to be considered when a company plans to work with, regenerate, and renew those ‘resources’ (Müller-Christ, 2010). Spillover effects between business and personal life are an important factor for sustainable growth, as they might affect e.g. health, engagement or qualification of the workforce. Furthermore, the workforce vice versa affects ecological and social environment like e.g. society or family, which should be included in any sustainability consideration (Ehnert, 2012; similar Zaugg, 2009).

With regard to this background, ‘Sustainable HRM’ has been defined by Ehnert (2009), p. 74 as “the pattern of planned or emerging human resource strategies and practices intended to enable organizational [and individual] goal achievement while simultaneously reproducing the HR base over a long-lasting calendar time and [while] controlling for self-induced side and feedback effects of HR systems on the HR base and thus on the company itself.”. This definition emphasizes the long-term, strategic character of the sustainability concept as well as the need to integrate the organizational and the individual perspective. If human resource management is “the process of acquiring, training, appraising, and compensating employees, and of attending to their labor relations, health and safety, and fairness concerns” (Dessler, 2011, p. 30), ‘Sustainable HRM’ is to achieve all this on a durably ongoing basis in a way that accounts for the economic, social and ecological needs of the organization, its members, and other stakeholders.

## Understanding of part-time leadership

In the following chapter, a clarification of the term ‘part-time leadership’ will be given. Furthermore, different part-time leadership working models will be introduced and predominance and acceptance discussed.

### Definition of part-time leadership

At first glance, the concept of part-time leadership seems self-evident. However, both of its components leave substantial room for interpretation (Karlshaus, 2016; Karlshaus & Kaehler, 2017a). Part-time work refers to a shorter than usual working time. As such, some sources use a fixed hourly contingent, e.g. the OECD definition of part-time employment refers to persons who usually work fewer than 30 h (Organisation for Economic Co-operation and Development (OECD), 2016; Van Bastelaer, Lemaître, & Marianna, 1997). Another source defines part-time work as subjective self-assessment (European Statistical System (ESS), 2016): Part-time work is recorded as self-reported by individuals. “The most common and convincing approach is to use the full-term standard as a reference: „… the term part-time worker means an employed person whose normal hours of work are less than those of comparable full-time workers “(ILO International Labour Organization, 1994; similarly, § 2 of the German “Teilzeit- und Befristungsgesetz” cf. Boecken & Joussen, 2012). However, according to this definition, part-time employment is a relative phenomenon. The full-time contingent in some organizations may well correspond to greatly reduced part-time arrangements in other organizations. For instance, Promberger et al. (1997) refer to the example of the 1994 Volkswagen initiative of 28.8 working hours per week. Also, from an international perspective, the average working time differs considerably, so that e.g. the level for a part-time employment amounts to 36 h in Germany, 35 h in the US, and only 30 h in Canada and the UK (Fagan et al., 2014).

The second element of the concept, leadership, also calls for clarification. As a process, leadership may be defined as „influencing others to understand and agree about what needs to be done and how to do it, and the process of facilitating individual and collective efforts to accomplish shared objectives. “(Yukl, 2013, p. 23). While some attempts have been made to distinguish managers from leaders (Bennis & Nanus, 1985; Kotter, 1990; Zaleznik, 1977), in the context of part-time leadership or part-time management this differentiation seems rather irrelevant, so that both terms may be used synonymously. A more decisive criteria for leadership is represented by the position of the job holder that is characterized by its responsibility for an organizational unit and particularly for the productivity and well-being of the people working in this unit. Productivity and well-being also constitute specific challenges in regard of management routines, work organization and availability, factors especially relevant for the arrangement of working time that is significantly different from mere responsible expert positions. Hence, the latter aspect of people responsibility is usually dominant. Thus, in the following the term ‘part-time leadership’ shall be used only for a description of the position of managers, board members and self-employed executives with disciplinary responsibility for at least three employees (Karlshaus & Kaehler, 2017a).

Part-time leadership is to be distinguished from the concept of ‚shared leadership‘, which describes the involvement of team members in leadership issues (Pearce & Conger, 2003; Perry, Pearce, & Sims, 1999). Whilst shared leadership is not in any way addressing reduced working time arrangements, it is rather about shifting management tasks in a cooperative full-time management environment to the team. Similarly ‚Co-leadership**‘**, which describes the joint performance of leadership tasks by a management team, is in most cases simply a matter of full-time managers working together (Alvarez, Svejenova, & Vives, 2007; Heenan & Bennis, 1999). Finally, part-time leadership must also be differentiated from concepts of ‚distributed leadership‘, which aim to expand leadership skills and enable or empower each individual team member to develop and demonstrate leadership competencies (Bolden, 2011). However, the basic premise of these concepts - according to which leadership can be a multi-person concept - undermines the traditional focus of leadership science and practice on a single full-time executive being a non alternative option. It thus opens up the scope for solutions in part-time leadership settings, because the joint leadership responsibility of leadership tandems or the stronger delegation of leadership tasks in distributed or shared leadership settings are ultimately based on the same mechanisms and illustrate the feasibility of part-time leadership concepts.

### Working time models in part-time leadership

Part-time leadership arrangements may include a wide variety of working time patterns from a few hours per week to nearly full-time standards. Also, the distribution of time can differ. There are models in which the reduced working time is distributed over all five working days or concentrated on fewer working days per week, so that whole free days can arise (Karlshaus, 2016). Part-time leadership models can be distinguished in three different types: (1) almost full-time part-time leadership models, (2) job sharing or job splitting models, and (3) cadre models (Karlshaus, 2016; Karlshaus & Kaehler, 2017a).

The most common approach – in particular with regard to higher management levels – is the almost full-time leadership model, in which the weekly working hours are reduced only minimally to 75% - 90% (Mogler, 2013). The distribution of working time can be both regularly or irregularly. When implementing this model, it is important to determine the regulations for overtime in advance as overtime arises frequently. Additionally, one has to prevent negative consequences for the team, e.g. increasing workload. Certainly, this model represents an advantage for companies because the absence of its managers is barely noticeable (Karlshaus & Kaehler, 2017a).

Job sharing, sometimes also referred to as top-sharing, means to split one job and the corresponding salary between two or more persons, each with their own contract of employment. This can be implemented by halving a full-time position, but the joint working volume can also be distributed unevenly (; BMFSFJ (Bundesministerium für Familie, Senioren, Frauen und Jugend) (Ed.), 1999; Domsch, Kleiminger, Ladwig, & Strasse, 1998; Karlshaus & Kaehler, 2017c; Kuark, 2002). The company benefits from this model as knowledge is secured in the company, a higher capacity in peak times is possible and creativity, motivation and productivity are increased. Also, absence management is much easier to handle by having two managers for one position (BMFSFJ (Bundesministerium für Familie, Senioren, Frauen und Jugend) (Ed.), 1999; Karlshaus & Kaehler, 2017a). Job splitting is similar to job sharing with the difference that two persons perform their specialist and managerial tasks independently of one another (Mogler, 2013). Thus, there is little need for interaction and cooperation between the two part-time managers, which means that there are also no conflicts on policies. However, knowledge protection and capacity expansion cannot be realized. Based on these facts, this model is recommended only for areas in which responsibility can be divided easily, e.g. for sales areas (Karlshaus, 2016).

The cadre model refers to a ‘deputy system model’ of highly qualified and trained managers. It can be applied with partners of equal hierarchy (see jobsharing) or with partners from different hierarchy levels. In the latter case, the manager reduces working time and works closely together with a newly appointed personal assistant. The costs of this new employee will be offset by a reduction of the manager’s salary. This model secures knowledge in the company and supports the recruitment of young professionals (Karlshaus, 2016; Karlshaus & Kaehler, 2017a).

### Predominance and acceptance of part-time leadership

Statistics on the proportion of part-time workers vary depending on the underlying definition of the hourly volume of a part-time worker. In addition, the definition of a manager can also vary depending on whether specialist, budgetary or personnel responsibility must be fulfilled as essential characteristics. However, it is a fact that the proportion of part-time leaders differs among individual countries in Europe.

A comparison of 30 countries in Europe shows those differences. Even though 30% of all employees in Germany work part-time, only 9% of those are in leading positions. With this percentage Germany is in the midfield of all countries. The highest percentage of part-time managers constitute Island and Malta with 22%, whereas Romania with only 1% represents the smallest share. The amount of female part-time managers is predominant in every country (Stuth & Hipp, 2017).

The aforementioned differences in the amount of part-time leadership between European countries can be justified by three explanations: Firstly, it depends on the common predominance of part-time work in general. If part-time work is quite popular in a national society, the number of managers in part-time employment increases as well. Secondly, it has been found that the higher the GDP of one country, the higher the number of part-time leadership positions. One explanation is that managers in wealthy countries do not only work to earn their livings but also for ‘self fulfilment’ or ‘self-realization’. Thirdly, the number of part-time managers depends on the existence of a legal claim for part-time work in this country. If the country provides a strong legal-claim, there will be especially more women, who then work in part-time leadership roles (Stuth & Hipp, 2017).

While in Germany every fourth female manager works part-time, the proportion of men only amounts to 4%. When considering industry-specific differences, it is striking that in the service sector part-time managers are employed more frequently than in other sectors. For example, in public administration, education and healthcare almost 30% of all female managers work in some kind of part-time position (Stuth & Hipp, 2017). However, the number of part-time executives depends not only on the industry, the proportion of women to overall workforce, and the proportion of general part-time employees (Stuth & Hipp, 2017), but also on the average age of the workforce, the number of hierarchical levels, and the corporate culture.

Generally, one can observe a rising trend of pro-actively offered part-time leadership programs in Germany. A significant number of German companies has been developing company specific part-time leadership concepts (Viering, 2009). An analysis of German DAX companies in 2015 and best practice cases show that those part-time leadership concepts and pilots mainly address flexible work hours and hourly reductions for a better coordination of work and family as well as a generation of higher quotas of female managers in upper leadership positions (Karlshaus, 2016; Karlshaus, 2018; Karlshaus & Kaehler, 2017d). Furthermore, it has been found, that even though in some companies official part-time leadership programs or initiatives have been in place, most companies are generally quite open-minded to develop individual part-time exceptions for managers that would like to reduce their working time due to family reasons (Karlshaus & Kaehler, 2017a).

Especially in the context of women’s networks as well as diversity and working time initiatives, part-time leadership has become a central topic, that is also gaining rising attention in the German society: 1000 companies have already been certified with the label “work and family” (“*berufundfamilie*”); the corporate network “Success Factor Family” (“*Erfolgsfaktor Familie*”) has reached 5000 members. Moreover, politics and economics strive towards innovative, modern and family-friendly working time models. A considerable number of companies have signed the “charter for family-aware working hours” (“*Charta für familienbewusste Arbeitszeiten*”) that has been promoted by the German chancellor Angela Merkel in 2011 (Karlshaus, 2016, p. 83).

## Theoretical framework: part-time leadership as instrument for sustainable HRM

In the following the suitability of part-time leadership as instrument for a sustainable HRM approach is discussed based on the theoretical frameworks of the ‘Triple Bottom Line’ to illustrate the interlinkage between part-time leadership and the three relevant subject areas of a sustainable management approach. Afterwards more specifically the impact of ‘part-time leadership’ on selected SDG indicators will be discussed.

### Part-time leadership as contributor to the triple bottom line

CSR typically addresses the dimensions ‘ecology’, ‘economy’, and ‘society’, that are referred to by the term ‘Triple Bottom Line‘(Elkington, 1999). The social line refers to the behavior of the company towards its labor and the community. This behavior should be fair and beneficial to the society, so that value is created for them. The economic line relates to organization’s business practices that create profit and therewith economic value. The environmental line refers to practices of the company that support the conservation of habitats (Elkington, 1999).

**Fig. 1:**
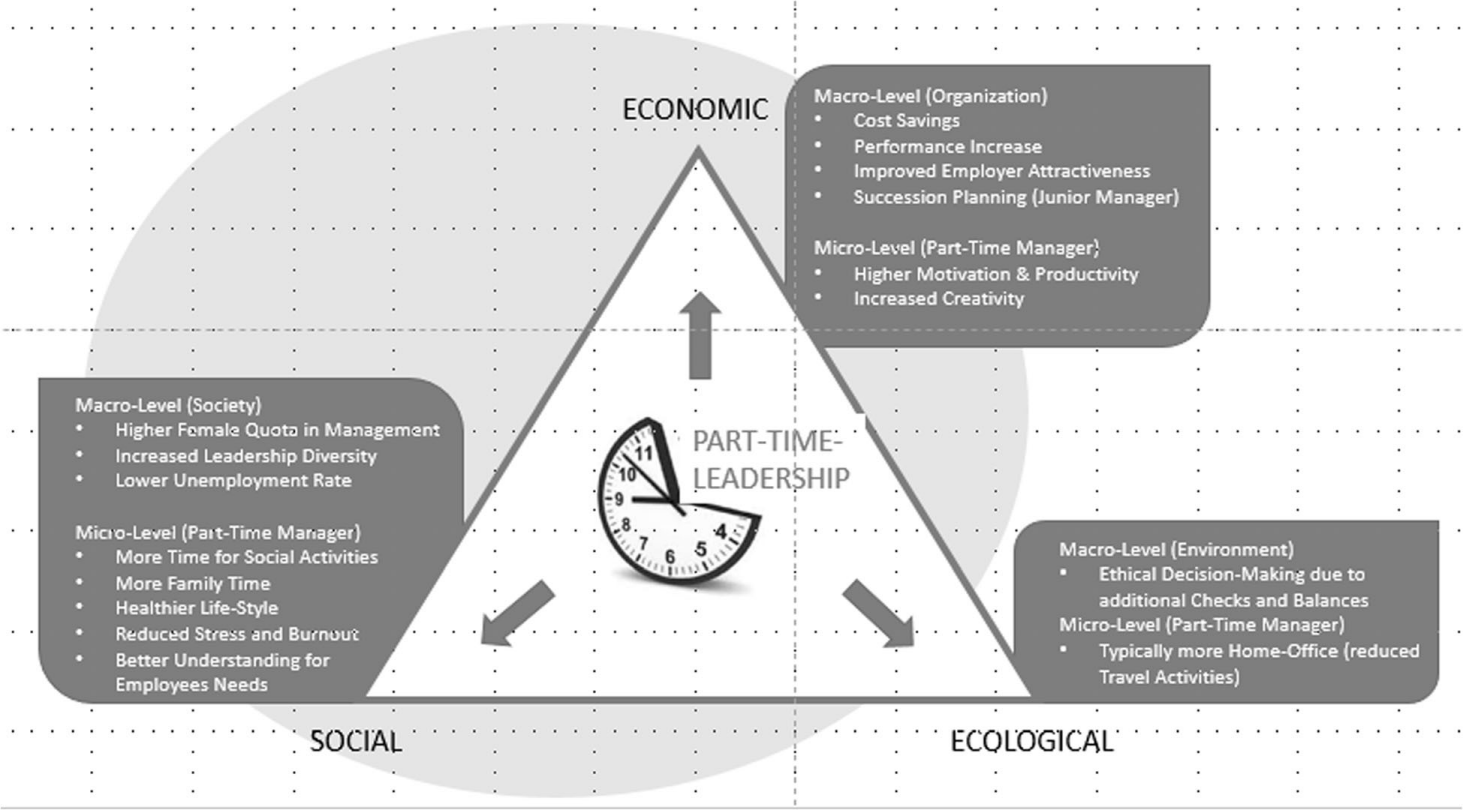
Part-Time Leadership affecting the CSR-Triangle

Organizational part-time leadership contributes in particular to the economical and social elements of the framework (Karlshaus, 2016), as it helps not only to reconcile the interests of the organization with those of the individual manager as an employee, but also with the larger interest of family members and society (see Fig. 1).

Concerning the *economic dimension* of the Triple Bottom Line, part-time leadership models create economic value due to their attractiveness for candidates. For many executives, being given the opportunity to balance e.g. ‘work and family time’ or ‘work and further education’ or ‘work and health related handicaps’ – while keeping a responsible leadership role – is very appealing. Therefore, part-time leadership models not only increase the employer attractiveness of a company with respect to current employees but also its competitiveness on the labor market. Furthermore, costs might be reduced to a lower sickness and reduced absenteeism rate, more task delegation to cheaper employees and a better degree of capacity utilization. Additionally, performance can be boosted due to a higher motivation, productivity and creativity of the part-time executives. Lastly, part-time leadership has been acknowledged as a useful and important instrument to encourage junior managers who might get more responsibility due to the reduced time factor of their part-time managers (Karlshaus, 2016; Karlshaus & Kaehler, 2017a).

Part-time leadership is also concerned with the *social dimension* of the ‘Triple Bottom Line’ mainly in a sense that managers have more time for their social activities. Since part-time employment in responsible positions is almost always voluntary (Karlshaus & Kaehler, 2017a), the interests of the manager are prevailing. In this context, part-time employment might help to liberate life time for other purposes, which often are connected to family needs or voluntary work. Even where spare time is used for recreation or private business activities, a social component is often involved. However, having more time for family, friends, and hobbies results in a better work-life-balance and a reduced likelihood of stress and burnout. Herewith managers might also develop a better understanding for the needs of their employees (Karlshaus & Kaehler, 2017a). On a societal level, part-time leadership models increase the number of women in leadership positions, as young mothers have still enough time to take care of their children and might decrease the unemployment rate respectively.

The impact of part-time leadership on the *ecological dimension* is limited. However, the travel activity of part-time executives can be minimized through a reduction of working days or increased appliance of home-office, telephone or video communication, which are often interlinked (Karlshaus & Kaehler, 2017a). In the case of job-sharing or job-splitting models, the mutual surveillance supports more ethical decision-making also with respect to ecological questions.

### Part-time leadership as contributor to selected sustainable development goals

In September 2015, the member states of the United Nations passed the Agenda 2030, an international framework for action. At the core of the agenda are 17 global sustainable development goals (SDGs) with 169 sub-goals (indicators) which represent a call for action to companies and individuals worldwide to protect the planet, ensure prosperity for everyone and end poverty until 2030. As economic, social and ecological aspects have been specified in concrete goals and measurable indicators with a universal validity, companies can plan their sustainable activities accordingly (UN Global Compact, n.d.-b). More specifically, the 17 SDGs aim to ensure that companies align their businesses according to the ten principles on human rights, labor, environment, and anti-corruption (United Nations, n.d.-c). As shown above, part-time leadership models help to achieve sustainability on all three dimensions of the triple bottom line. By implementing part-time leadership models, companies can also address some of the SDGs – even if such a leadership concept is mainly valid for the Western world or highly developed countries and SDG indicators have to be interpreted and applied accordingly in a local context.

In the following, Sustainable Development Goals No 3, 5, and 8 are being discussed with respect to part-time leadership and its link to sustainability as they show the greatest overlap concerning the goals and benefits of both concepts. In addition, they highlight very well the broad range of effects part-time leadership models can have on sustainable HR management. Existing qualitative and quantitative studies as well as best practice examples on part-time leadership are being presented to illustrate existing implementation strategies and measures.

#### SDG goal 3: the third SDG aims to “ensure healthy lives and promote well-being for all at all ages” (United Nations, n.d.-a)

Part-time leadership has a positive effect on the health of the respective employees. It reduces the likelihood of work overload as well as stress, and therefore helps prevent burnout (Dellekönig, 1995; Hinz, 2008). For example the travel company, TUI GmbH, describes this wish for working time flexibility with the term ‘working time sovereignty’, and shows the necessity of companies to not ignore the needs of their employees and managers (Meyenberg & Schinner, 2017) in order to keep them healthy and productive. The key of part-time leadership models is basically its flexibility which leads to a better balance of work, family and friends, health and values (Karlshaus, 2016). It thereby reduces the burden for executives which arises e.g. from child care, care of elderly or sick persons, parallel undertaken training programs or voluntary commitment which might in excessive extent otherwise lead to stress, addictions or burnout (Puls, 2013). In this sense, part-time management can help to address indicator 3.5 “Strengthen the prevention and treatment of substance abuse, including narcotic drug abuse and harmful use of alcohol” (United Nations, n.d.-a). In addition to this a positive impact on indicator 3**.**D “Strengthen the capacity of all countries, in particular developing countries, for early warning, risk reduction and management of national and global health risks”) **can be assumed** (United Nations, n.d.-a). This is especially true if considering “burnout” a potential national disease of developed countries (Lövelt, 2013). Additionally, part-time leadership models also strive for a well-being of elderly employees, also called ‘silver workers’ (Lechner, 2017). In the case of the insurance company Barmenia, part-time leadership has been guaranteed to facilitate the transition into retirement by allowing the elderly manager to already start to establish new (private) tasks and responsibilities outside of the company (Rüß, 2017) in order to guarantee healthy aging. Time reduction not just supports a sustainable ‘life phase orientation’, but furthermore helps to prevent overwork-driven diseases.

#### SDG goal 5: the fifth SDG aims to “achieve gender equality and empower all women and girls” (United Nations, n.d.-b)

Part-time leadership can be regarded as an instrument which fosters gender equality, since it allows more women with family obligations to perform a leadership role. Therewith, it balances the distribution of power in organizations (Meyenberg & Schinner, 2017). In this sense, part-time management directly addresses indicator 5.5 **“** Ensure women’s full and effective participation and equal opportunities for leadership at all levels of decision-making in political, economic and public life.**”** (United Nations, n.d.-b), being more specifically defined in indicator 5.5.2 ‘Proportion of women in managerial positions’ – but also valid for 5.5.1. ‘Proportion of seats held by women in national parliaments and local governments‘. One of the main reasons for executives to decide for a part-time role is the better compatibility of work and family. These leaders will also have a greater understanding for their employees’ wishes concerning family time which serves young female talents in particular (Jochmann-Döll, 2017). Several companies use flexible working time models to improve gender equality as some of the following examples might elucidate. The automotive company, Daimler AG, implemented individual, diverse and flexible working time models with the objective to have at least every fifth position filled with a female executive by 2020 (Lechner, 2017). Also, the company Ernst & Young designs personalized and flexible working time models resulting in an increasing number of part-time employees and women in leadership positions (Galvagni & Voß, 2017). The insurance company HUK-Coburg also engaged in the development of individual part-time positions, especially for previous female executives who return from parental leave (Rössler & Renning, 2017). Furthermore, the City of Cologne is highly committed in consequently developing equal opportunity programs for women and thus successfully implemented ‘close to fulltime leadership’ possibilities on all management levels with the result of a rising female quota (Kronenberg, 2017). Finally, a study on behalf of the Hans-Böckler-Foundation with the research question to analyze part-time leadership at the police in Germany found a significant correlation between the number of part-time managers, their performance evaluations and the female quota in the various management levels (Jochmann-Döll, 2017). The higher the proportion of part-time managers, the more similar the average employee ratings are to full-time managers. As long as part-time management is an atypical management concept, they tend to be rated worse. Since part-time management is largely carried out by women, it can be concluded that a critical mass of part-time management in a company also serves the indicator 5.1 “End all forms of discrimination against all women and girls everywhere” (United Nations, n.d.-b).

#### SDG goal 8: the eighth SDG aims to “promote inclusive and sustainable economic growth, employment and decent work for all” (United Nations, n.d.-c)

A part-time leadership model is an instrument for securing skilled employees as it enables more people to work and balance work and private life. By offering part-time management, new groups of employees such as women are addressed and access to these employees is facilitated from a company point of view, thus increasing diversity and thereby achieving greater economic productivity (McKinsey, 2018). This effect is corresponding to indicator 8.2. “Achieve higher levels of economic productivity through diversification, technological upgrading and innovation, including through a focus on high-value added and labour-intensive sectors” (United Nations, n.d.-c.). Additionally, more responsible decisions will be made due to a stronger control in job-sharing concepts on leadership level - but also through delegation and jointly made decisions (Karlshaus, 2016). Furthermore, such a part-time model can increase performance and productivity through highly concentrated work results of executives or the compression of working time of part-time employees, but also due to a rise in creativity, motivation and innovation of part-time executives (Karlshaus & Kaehler, 2017a, b, c, d). Such effects have been described e.g. by the consultancy EY (Galvagni & Voß, 2017). Moreover, part-time leadership models can support youth development because the part-time executive has to involve high potentials into management tasks to a higher extent (Mogler, 2013). Furthermore ‘part-time leadership’ might support indicator 8.5 “By 2030, achieve full and productive employment and decent work for all women and men, including for young people and persons with disabilities, and equal pay for work of equal value” (United Nations, n.d.-c.) as it might decrease unemployment by redistributing leadership tasks on possibly more shoulders. Furthermore, also handicapped or sick people would have a fair chance on management positions as they might not be able to work full-time. Additionally, ‘part-time leadership’ models can reduce personnel costs and therefore support organizational efficiency-campaigns (Seifert, 2008). Costs can be saved due to the fact that tasks from the rather expensive executive are delegated to the rather cost-effective employee as the example of the financial institution Commerzbank shows (Bauernfeind, Prößl, & Warkus, 2017).

## Critical success factors for implementing part-time leadership

When considering implementing a part-time leadership model, one should be prepared for several barriers which could complicate the successful implementation. To meet these challenges, the macro, micro, and process perspective should be respected. The macro perspective includes the corporate culture, structure and strategy, while the micro perspective contains detailed aspects of the task content and the respective actors, called the ‘part-time-family’. The process perspective is represented by the implementation of part-time leadership on the corporate level and in individual cases (Karlshaus & Kaehler, 2017a). In the following, the individual sub-chapters will analyze the potential causes and barriers of successful part-time leadership and will outline potential solutions.

### Macro perspective: culture, structure, strategy

Barriers which may hinder the successful implementation of part-time leadership models can lie in culture, structure and strategy of the company.

With respect to *corporate culture*, part-time leadership requires a high degree of trust, result orientation, and openness and innovation in the company (Mogler, 2013). Unfortunately, part-time executives are sometimes considered to be less committed, motivated, flexible and career-oriented, in particular if a so-called ‘presence-culture’ is predominant (Trost & Wagner, 2002; Vogel, 2006). Due to such prejudices, part-time executives may experience disadvantages as e.g. the exclusion from certain networks and circles or important meetings that cannot be realized due to the time constraints of a part-time manager (Vedder & Vedder, 2008). Additionally, the probability of promotion, bonuses and training days often decreases (Vedder & Vedder, 2008). Therefore, it is recommended to integrate part-time leadership into the corporate culture and to try to mitigate micropolitical disadvantages. This can be realized by applying typical change management instruments, e.g. communication and information campaigns, top-management support, formal part-time-leadership initiative pilots, etc. Network meetings, multiplier workshops, or even trainings on the topic serve to convey positive aspects of part-time leadership (Karlshaus, 2016).

The implementation of part-time executive positions at management level affects established *work processes* and traditional workplace structures (Vedder & Vedder, 2008). In this context, nontransparent and inflexible processes as well as limited possibilities of operationally handling part-time leadership within the existing IT landscape are to be named as typical barriers. Therefore, proactive and direct communication channels between employees, part-time managers, management, and customers are of central importance. Suitable organizational structures are characterized by a high degree of decentralization, flat hierarchies and a high degree of employee autonomy (Karlshaus, 2016). Thus, it is recommended to create transparent, flexible and preferably decentralized structures with a high degree of employee autonomy. Particularly important at management level is the agreement of flexible working time models, in which organizational requirements can be encountered situationally and attendances are not fixed rigidly in advance. It is important for the employees that they are allowed to act autonomously to a certain extent. In the best case, persons with a direct connection to management and the executive board should determine the framework for part-time leadership positions (Karlshaus, 2016).

According to the *corporate strategy*, one can say that a strategy for equal opportunities as well as an innovation strategy certainly favors the implementation of part-time leadership concepts. However, a restructuring policy may focus more on cost aspects, which make part-time management and job-sharing models seem less important. While politicians, the public, the media and many employees themselves often can imagine a reduction in working hours very well, such models may not correspond to the interests and lobby of customers, lenders, and suppliers. Customers often demand, for example, a high level of service, flexibility and responsiveness, personal proximity as well as high value for money. These parameters are diametrically opposed to the limited presence and accessibility of part-time management (Karlshaus, 2016). For a successful implementation, it is recommended to increase the relevancy of working time flexibility and reduction in the corporate strategy and to involve stakeholder groups in persuasion. Examples prove that a three-day presence with the client is endorsed and accepted, if there is a clear and transparent communication on attendance rules and a certain accessibility on the remaining 2 days is ensured. In addition, it is important to position part-time management as the most important aspect of a family-friendly corporate strategy. Precise targets such as a 10% part-time leadership position by 2022 are helpful (Karlshaus, 2016).

### Micro perspective: ‘part-time-family’ and task content

Furthermore, the so-called ‘part-time-family’ and the right task content are critical factors which enhance a successful part-time leadership role.

With respect to the *task content*, it is necessary to analyze the variety and quantity of tasks that are adequate for the respective position. Suitable tasks for a part-time management position are more standardized, predictable, less time-critical, and less complex. Internal and external customers as well as adjacent areas ideally require a non-daily personal communication effort (Karlshaus, 2016). In respect to the delegation of tasks, contents such as reporting, participation in day-to-day business or partially conceptual tasks are generally considered appropriate (Kratzer & Neidl, 2011). In contrast, classic disciplinary management tasks, which include e.g. target agreements, target assessments, employee appraisals, or recruiting are generally not suitable for delegation, but are primary leadership duties. Due to the fact that jobs for part-time managers usually result from a reduction of a full-time position, the problem arises that sometimes the working time and earnings are reduced ‘officially’ but the workload remains the same (Vedder & Vedder, 2008). A clear task description and an adequate reduction of the workload is recommended to secure a successful implementation. The preparation of differentiated and transparent task and job descriptions is suggested as well as a distinction between delegable and non-delegable work place components (Kaehler & Karlshaus, 2014). To determine the adequate amount of work for the corresponding part-time position, it may be necessary to include the involvement of part-time managers with correspondingly extensive experience.

The so-called ‘*part-time-family’* consists of the part-time executive’s employees, supervisor, colleagues, clients, etc. For a successful implementation, it is essential that all actors in this family understand the working scopes and accessibilities of the part-time manager and support this working time model (Fauth-Herkner & Leist, 2001). Otherwise, the motivation and satisfaction of the executive as well as of his team may be impacted negatively. Additionally, a bad preparation for this role could result in a higher workload for the employees, as former tasks of the executive are just passed to his or her employees, which is counterproductive for the acceptance of part-time leadership in general. With regard to the ‘part-time-family’, it is recommended to inform and integrate all actors in the planning and implementation progress to prevent possible conflicts. To support the employees of the part-time executive, one should offer training, coaching or mentoring to improve their abilities of self-management, autonomy and self-responsibility. The HR consultants need to be well informed and have to evaluate the process regularly, so that they can develop and adopt individual models, implement them successfully, and advise the executive during the conversion process (Karlshaus & Kaehler, 2017a).

### Process perspective: implementation of part-time leadership

Another critical success factor is the implementation process. The approach can either be an operational, company-wide model or an implementation on a case-by-case basis. When implementing an operational part-time leadership model, it is recommended to make use of a pilot project and to support the process with communication events. Additionally, it is important to elaborate on prejudices and find solutions for possible disadvantages, such as fewer bonuses and less promotions (Karlshaus & Kaehler, 2017a). When implementing an individual case, one should analyze the needs of the executive in the beginning and discuss the individual process steps. Also, it is recommended to offer informative events. Additionally, the executive as well as his or her employees should receive training, coaching or mentoring. Generally, the model should be very flexible and controlled regularly (Karlshaus & Kaehler, 2017a).

## Summary and conclusion

Despite an increasingly open-minded approach to part-time leadership, there currently is still a comparatively low rate of only 9% of part-time executives in Germany mainly female and in the lower to middle management. This fact can be largely explained by corporate structural, cultural, and strategic parameters: structures are not sufficiently aligned with part-time work, there are existing acceptance problems among different stakeholder groups, and from the perspective of the part-time manager there is a lack of equal opportunities in terms of promotion, training, and remuneration. As a result, part-time work is still not fully recognized as an alternative and equivalent working-time arrangement for executives. Part-time leadership however represents an important opportunity for organizations especially in the context of sustainability.

In this context, a number of theoretical and practical implications can be derived from the article. Based on the theoretical model of Elkington, the article highlights how part-time leadership addresses all three dimensions of the ‘Triple Bottom Line’ and demonstrates how substantial the intersection between the concepts of ‘part-time leadership’ and ‘CSR’ is. For this purpose, a theoretical explanation and definitions of the underlying concepts have been provided, which lead to a better understanding of ‘part-time leadership’ in particular. The article has thus applied and extended the theoretical concepts of ‘part-time leadership’ and sustainability as it reveals the mutual impact by systematically combining the underlying purposes and goal objectives.

Furthermore, the following practical implications are to be pointed out: By implementing part-time leadership models, a company increases its employer attractiveness by improving its work-life balance situation and as a result gains new qualified employees. Employees are not only more satisfied, motivated, and creative, but also less stressed and healthier, which is reflected in higher productivity and lower absence rates. Furthermore, companies might save costs, develop junior managers in a smart way and foster a more human and understanding management style that might lead to a higher wellbeing of all affected employees. Finally, it encourages gender equality within the company. The challenge is that there are no standard or patent solutions for the successful development and implementation of part-time leadership models that also could serve sustainability goals. Implementing part-time leadership requires individual solutions, which have to take into account the task, the team, the direct supervisor, the part-time manager, the customer, and other external conditions. Important in the development of any atypical working time model is the consideration of the identified barriers and solutions. These need to be mastered systematically, for example by clearly delineating management tasks, promoting autonomy and flexibility, communicating relevant values and creating well defined rules and cooperation policies within the company. To help organizations overcome this implementation challenge, the article not only presents a high level overview of part-time leadership models and key success factors to consider for implementation, it also offers specific examples on how to link part-time leadership solutions to selected specific SDGs. The implementation of part-time leadership will help achieve the Sustainable Development Goals as demonstrated with several best practice cases of multinational companies, thereby clearly highlighting the role of part-time leadership as driver for sustainable HRM.

Although this article delivers some valuable conceptual ideas about the use of part-time leadership as instrument for a sustainable HR management as well as driver of selected SDGs, there are certainly a couple of limitations and shortcomings to be mentioned. First of all, the empirical research base is based on a selection of qualitative best practice cases. An additional quantitative approach to systematically capture barriers, benefits and success factors for implementation of part-time leadership as CSR instrument is advisable. Second, the concept and emergence of part-time leadership is very heterogeneous and context specific. Thus, an international or Western approach is accordingly imperfect and some statements might not be generalizable. Third, when considering the topic ‚part-time leadership‘, a multi-perspective approach should be taken by regarding the different stakeholder groups as well as company and individual part-time manager perspective.

Further, theoretical deep-dives in analyzing effects on the part-time managers‘ team or e.g. consequences of health related issues that also include psychological phenomena like stress or mobbing due to the unusual management profile, should be considered. In addition, further research on the effect of part-time leadership on gender equality is recommended as such an atypical working time model might also turn out as career obstacle in case company culture is requesting a high level of management attendance (‘presence culture’). Empirical research could also explore under which conditions companies choose to implement part-time leadership and explore the question to what extent part-time leadership has a positive impact on the company’s success. Last but not least, in the context of ‘part-time leadership’, the role of new technologies and tools that can support these leadership models should also be examined more closely. Similarly, the integration of part-time leadership into the “Future of Work” framework or the analysis of the Covid-19 implications as a potential ‘accelerator’ of the topic would be an interesting subject for further research.

However, there are already ‘new’ facilitating factors that accelerate the design and implementation of part-time leadership approaches today, such as a significant shortage of skilled workers in some professions, a high and more and more relevant proportion of female and young employees in the workforce, as well as a value shift towards a higher demand for work-life balance concepts. Moreover, there are new possibilities for implementation through innovative planning and information technologies, appropriate legal frameworks, a whole series of ‘best practice examples’ as well as an already noticeable gain in acceptance in Europe. Nevertheless, part-time leadership will predominate in companies only if it is managed to create a vibrant and serious culture of flexible working time and develop appropriate strategies and structures to reach a sustainable business transformation towards more individual and life phased oriented working time arrangements. Modern, flexible or shorter working models – especially in the management sector – are to be regarded as a key issue of a future-oriented sustainable HRM policy. Against the background of today’s demarcation of work and private life, the introduction of part-time leadership models is a component of a more sustainable world as it has the potential to significantly relieve the society, current and future management generations as well as the economy, thus creating a win-win-win situation.

## Data Availability

Not applicable.

## Abbreviations

CSR: Corporate social responsibility
HR: Human Resources
HRM: Human Resources Management
ILO: International Labour Organization
OECD: Organisation for Economic Co-operation and Development
SDG: Sustainable Development Goals
